# Adjuvant treatment of gastrointestinal stromal tumors (GIST)—state of the art, controversies, and future directions

**DOI:** 10.1016/j.esmogo.2026.100397

**Published:** 2026-08-24

**Authors:** A.-M. Scheuble, F. Lordick, R.G. Maki

**Affiliations:** 1 Department of Medicine (Oncology, Gastroenterology, Hepatology, Pulmonology), University of Leipzig Medical Center, Comprehensive Cancer Center Central Germany (CCCG), Leipzig, Germany; 2 Department of Medicine, Memorial Sloan-Kettering Cancer Center, New York, USA

**Keywords:** adjuvant, gastrointestinal stromal tumor, risk stratification, tumor rupture

## Abstract

Adjuvant treatment of gastrointestinal stromal tumors (GISTs) with imatinib has improved recurrence-free survival in patients at high risk of relapse after complete surgical resection. Nevertheless, its impact on overall survival (OS) remains limited, and optimal patient selection and treatment duration remain subjects of ongoing debate. Current risk stratification relies on tumor size, mitotic count, anatomical location, and tumor rupture, yet these factors incompletely capture the biological heterogeneity of GISTs. In particular, intermediate-risk disease represents a heterogeneous group for which the benefit of adjuvant therapy remains uncertain and requires individualized decision-making. Molecular profiling has become an essential component of GIST management. Mutations in KIT and platelet-derived growth factor receptor alpha (PDGFRA) provide important prognostic and predictive information. KIT exon 11 mutations are associated with the greatest sensitivity to imatinib, whereas exon 9 mutations and non-KIT/PDGFRA GISTs exhibit distinct biological behavior and therapeutic responses. Tumor rupture is consistently associated with a high risk of recurrence, although optimal treatment strategies are not clearly established. Three years of adjuvant imatinib is currently considered the standard of care for high-risk GISTs. Nevertheless, recurrence frequently occurs after treatment discontinuation, particularly in very high-risk patients. Emerging evidence suggests that prolonged therapy may improve progression-free survival in selected subgroups, although a clear OS benefit has not been demonstrated. Future advances in risk stratification are expected to integrate molecular, multiomics, and artificial intelligence-based approaches to better identify patients most likely to benefit from adjuvant therapy, personalize treatment duration, and minimize overtreatment in localized GISTs. A comprehensive literature review was conducted using PubMed and major oncology congress resources. The search focused on English-language studies investigating adjuvant therapy duration, risk stratification, mutational profiling, and long-term outcomes in localized GISTs. Priority was given to randomized phase II/III trials, pivotal observational studies, and current international guideline recommendations.

## Highlights

• Adjuvant imatinib improves RFS in high-risk GIST after resection.
• OS benefit of adjuvant imatinib remains limited despite prolonged therapy.
• Molecular profiling refines prognosis and predicts imatinib sensitivity in GIST.
• Intermediate-risk GIST is heterogeneous and requires individualized treatment decisions.

## Introduction

Gastrointestinal stromal tumors (GISTs) are rare malignant mesenchymal tumors of the gastrointestinal tract, with an incidence rate of 10-15 cases per million people per year, broadly similar in Europe and the United States.^1^ Most GISTs harbor an activating driver mutation in *KIT* or platelet-derived growth factor receptor alpha (*PDGFRA*), allowing for a targeted therapeutic approach with tyrosine kinase inhibitors (TKIs) in both the adjuvant and metastatic settings.

Surgery is the most effective treatment for localized GISTs, but metastatic risk can be 70% or more after radical surgery for the highest-risk primary tumors, usually involving the liver and the peritoneum.^2^ Imatinib reduces the risk of relapse in patients with high-risk GIST, but the benefit to recurrence-free survival (RFS) in the adjuvant setting is more modest, in particular with extended follow-up.^3^^,^^4^ Despite imatinib’s efficacy, questions remain about the optimal duration of therapy, emphasizing the importance of precisely identifying patients at very high risk of recurrence.

## Moving toward more personalized therapy

Previous risk-stratification models incorporate established clinicopathological risk factors such as tumor size, mitotic count, anatomical location, and tumor rupture and are effective in distinguishing between low- and high-risk GISTs.^3^, ^4^, ^5^, ^6^, ^7^ The modified National Institutes of Health (NIH) criteria and the Armed Forces Institute of Pathology classification are widely used to categorize patients into very low-, low-, intermediate-, and high-risk groups. High-risk GIST is generally defined by large tumor size, high mitotic activity, and nongastric location. Tumor rupture is also included in the modified NIH criteria and is associated with a high risk of recurrence.^3^ Intermediate-risk disease (defined as either 2-5 cm tumors with >5 mitoses/50 high-power field or 5-10 cm tumors with ≤5 mitoses/50 high-power field) represents a biologically heterogeneous subgroup with variable recurrence risk.

A more refined classification can be achieved by using contour maps or nomograms. These tools employ continuous rather than dichotomized variables for tumor size and mitotic count. They are effective in predicting individual recurrence rates and can help to improve the ability to identify genuinely higher-risk patients.^4^, ^5^, ^6^ Using data from more than one method can be helpful for individual prognosis stratification.

Due to the favorable prognosis for low-risk GIST, adjuvant therapy is not beneficial and therefore not employed. The intermediate-risk group is more heterogeneous, and the role of imatinib in treating these patients is still evolving. Adjuvant therapy in patients with intermediate risk was addressed in two randomized phase III studies. The European Organisation for Research and Treatment of Cancer (EORTC) 62024 study did not demonstrate a benefit of 2 years of adjuvant imatinib according to the NIH risk criteria.^8^ In contrast, the ACOSOG Z9001 trial, which included patients with GIST >3 cm and evaluated 1 year of adjuvant imatinib, showed an improvement in RFS, but no overall survival (OS) benefit.^9^ Furthermore, the single-arm phase II PERSIST-5 study reported no recurrences among patients who completed 5 years of continuous imatinib therapy.^10^ Given these findings, decisions regarding adjuvant treatment in intermediate-risk GIST should be individualized and take into account factors such as tumor rupture, mutational profile, and patient preferences within a shared decision-making process (Table 1).

**Table 1:** Comparison of ESMO and NCCN guidelines of adjuvant imatinib in localized GIST

Clinical consideration	ESMO	NCCN
Duration of adjuvant therapy	3 years standard for high-risk GIST. Extension beyond 3 years may be considered in very high-risk patients, but is not universally recommended	3 years standard for high risk. Individualized extension may be considered, particularly after neoadjuvant therapy or in very high-risk cases
Risk stratification for adjuvant therapy	Strictly risk-based using modified NIH/AFIP criteria. Adjuvant therapy recommended only for high-risk disease. Intermediate risk generally not routine. Tumor rupture = high risk	NIH/AFIP-based risk assessment. High risk recommended. Intermediate risk may be considered based on clinical context and shared decision-making. Tumor rupture = high risk
Imatinib dose in *KIT* exon 9 (localized setting)	400 mg daily standard in adjuvant setting; 800 mg established in metastatic disease; not routinely recommended in the adjuvant setting	400 mg daily standard adjuvantly; 800 mg recommended primarily for metastatic/progressive disease

AFIP, Armed Forces Institute of Pathology; ESMO, European Society for Medical Oncology; GIST, gastrointestinal stromal tumor; NCCN, National Comprehensive Cancer Network; NIH, National Institutes of Health.

Molecular analysis confirming a *KIT* mutation is mandatory in patients who are candidates for adjuvant imatinib. The prognostic importance of various *KIT* mutations is well described.^11^ The most common *KIT* exon 11 mutations typically demonstrate high sensitivity to imatinib.^12^ Additionally, molecular sequencing identifies *KIT* exon 11 deletions, particularly those involving codons 557 and/or 558, as strong predictors of aggressive tumor behavior, higher recurrence risk, and poorer RFS compared with other mutations or wild-type GISTs.^13^^,^^14^ More aggressive clinical behavior, a higher risk of recurrence, and lower imatinib sensitivity are associated with *KIT* exon 9 mutations as well.^12^^,^^15^^,^^16^ Although the superiority of an 800-mg dose in the adjuvant setting, as opposed to the metastatic situation, has never been demonstrated in prospective randomized studies, some experts recommend the higher dose for *KIT* exon 9 mutations based on retrospective studies (ESMO Scale for Clinical Actionability of Molecular Targets score I-A),^17^, ^18^, ^19^ despite lack of benefit of 400 mg daily as noted in the subset analysis of patients treated on Scandinavian Sarcoma Group (SSG)-XVIII.

None of the randomized adjuvant trials selected patients based on their mutational profile. Nevertheless, retrospective subgroup analyses revealed a benefit of adjuvant treatment for patients with *KIT* exon 11 mutations, demonstrating improved 10-year RFS and OS.^12^ No advantage was demonstrated for other established mutations,^10^^,^^20^ with poorer outcomes noted overall regardless of therapy duration for *KIT* exon 9-mutated GIST. Incorporation of other genomic factors will help better stratify the risk-reward decision for adjuvant imatinib.

A recently published large retrospective cohort study demonstrated that adjuvant imatinib was associated with improved RFS and OS in patients with resected GISTs harboring KIT exon 9 mutations, particularly in high-risk disease.^21^ Additionally, no difference was observed between the 400 and 800 mg/day dosages. These results support adjuvant imatinib for patients with high-risk KIT exon 9-mutated GIST and indicate that dose escalation in the post-operative setting does not provide additional benefit.

## Tumor rupture

Tumor rupture occurs in 10%-27% of high-risk GISTs, and the recurrence rate for ruptured GISTs is 68% on average, irrespective of adjuvant treatment.^22^ Although tumor rupture is a risk-stratification criterion for adjuvant therapy, there is no widely accepted definition, as noted below.^3^^,^^6^^,^^23^ Similarly, the utility and duration of adjuvant therapy remain uncertain. For instance, the National Comprehensive Cancer Network guidelines equate tumor rupture with metastatic disease.^24^ Nevertheless, hemoperitoneum or bowel perforation without overt seeding into the peritoneal cavity or iatrogenic peritoneal damage during surgery does not substantially affect the recurrence rate.^23^^,^^25^

Based on the proposed definition of the Oslo Sarcoma Group, retrospective data contain six different clinical scenarios, including minor or major ruptures, representing the wide spectrum of rupture with potentially different consequences.^25^^,^^26^ Although this classification represents a step toward a standardized definition of tumor rupture, no prospective data on therapeutic implications are available yet.

In SSG-XVIII, patients with ruptured GIST did not benefit from 3 years of adjuvant imatinib compared with 1 year in terms of OS, although a high risk of relapse was observed after stopping adjuvant treatment.^27^ A subgroup of patients with ruptured GIST in the cohort of *KIT* exon 11 ins/del mutations had significantly better OS with 3-year adjuvant imatinib than those with 1-year treatment [hazard ratio (HR) 0.09, 95% confidence interval (CI) 0.01-0.74, *P* = 0.016]. These data indicate that mutation profiles are important for both ruptured and grossly intact GISTs. On the contrary, these data indicate that most patients in this situation will ultimately end up on imatinib lifelong.^28^^,^^29^ The two most recent studies on prolonged imatinib treatment in the adjuvant setting—the randomized IMADGIST trial and the single-arm PERSIST-5 trial—did not provide data that longer imatinib exposure improved survival.^10^^,^^30^

## NON-*KIT*/NON-*PDGFRA* GIST

Approximately 5% of GISTs examined in contemporary series lack *KIT* or *PDGFRA* alterations^31^^,^^32^ and are less sensitive to imatinib and subsequent TKIs.^33^ The *PDGFRA* D842V mutation is also associated with primary resistance to imatinib. Imatinib is also ineffective in succinate dehydrogenase-deficient GISTs due to its distinct oncogenic mechanism. The so-called succinate dehydrogenase subunit B-competent cases that are *KIT* and *PDGFRA* mutation-negative should be analyzed by next-generation sequencing to identify other potential targetable alterations. Adjuvant imatinib is not recommended for these GIST subtypes.^34^

## Optimal duration of adjuvant imatinib

**Figure 1:**
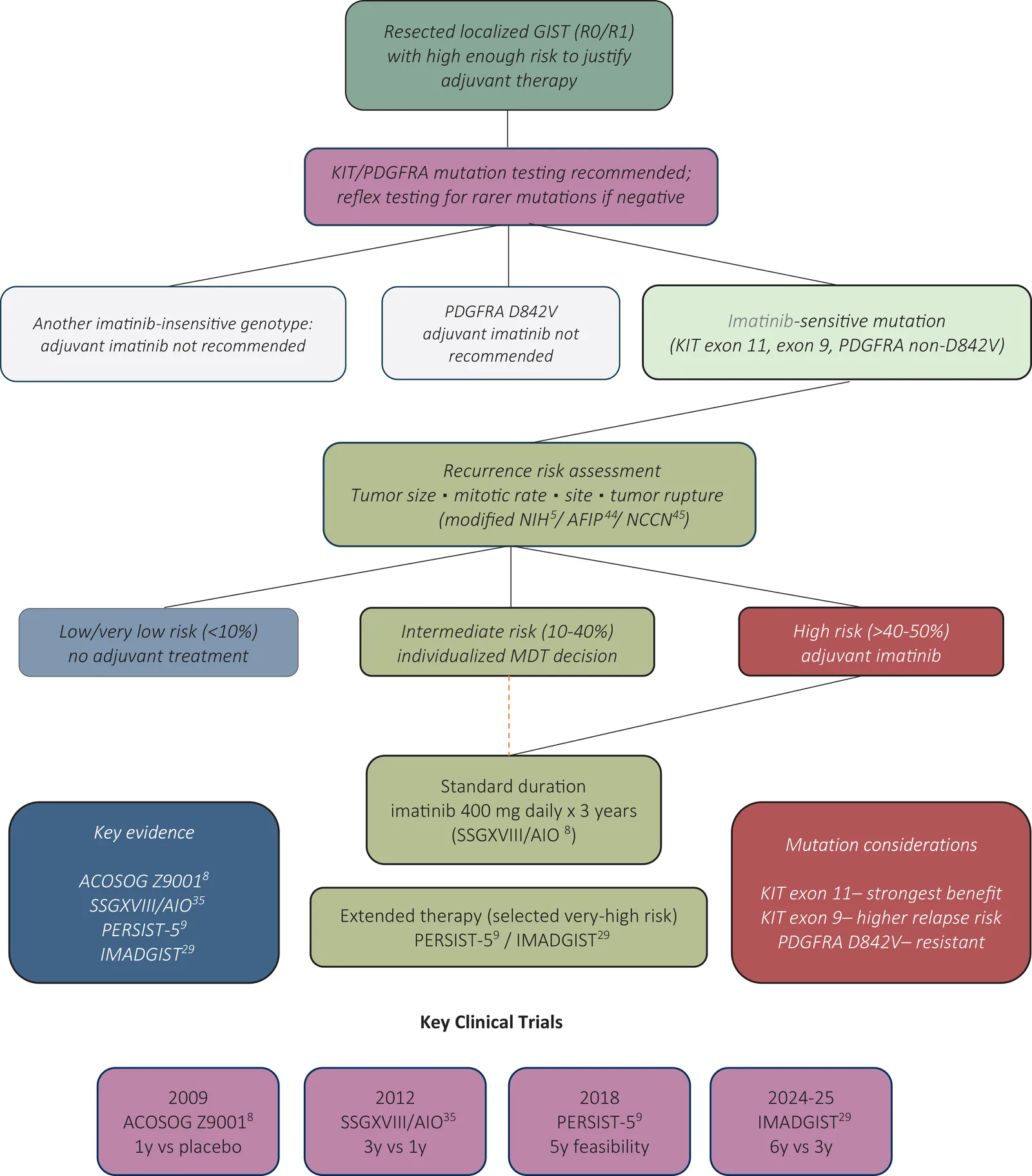
**Indication and duration of adjuvant imatinib in resected GIST**. AFIP, Armed Forces Institute of Pathology; GIST, gastrointestinal stromal tumor; MDT, multidisciplinary team; NCCN, National Comprehensive Cancer Network; NIH, National Institutes of Health; PDGFRA, platelet-derived growth factor receptor alpha.

Pivotal trials showed improved RFS for a longer duration of adjuvant treatment of patients with a higher risk of relapse (Table 2). Not surprisingly, these trials differed in terms of risk classification, inclusion criteria, and duration of adjuvant therapy.^9^^,^^37^ The only study to confirm better OS was the SSG-XVIII trial, which compared 1 versus 3 years of adjuvant imatinib.^35^ Since then, adjuvant therapy for 3 years has been considered standard for patients with higher-risk GIST (Figure 1).

**Table 2:** Key prospective and retrospective studies evaluating adjuvant imatinib therapy in localized GIST

Trial	Population	Study design	Treatment duration	Main findings
ACOSOG Z9001^9^	Resected GIST >3 cm	Phase III randomized trial	1 year versus placebo	Improved RFS, no OS benefit
EORTC 62024^8^	Intermediate- and high-risk localized GIST (NIH criteria)	Phase III randomized trial	2 years versus observation	No significant OS advantage; longer imatinib failure-free survival
SSG-XVIII/AIO^35^	High-risk resected GIST	Phase III randomized trial	3 versus 1 year	Improved RFS and OS with 3 years
SSG-XVIII/AIO (10-year follow-up)^36^	SSG-XVIII/AIO (10-year follow-up)	Long-term follow-up analysis	3 versus 1 year	Durable RFS and OS benefit maintained at 10 years
PERSIST-5^10^	Intermediate-/high-risk GIST	Single-arm phase II trial	5 years single-arm	No recurrence during treatment completion
IMADGIST^30^	High-risk localized GIST	Phase III, randomized trial	6 versus 3 years	Improved DFS and OS data remain immature
Kang et al.^29^	Ruptured localized GIST	Retrospective cohort	5 versus 3 years adjuvant imatinib	Longer treatment associated with improved RFS
Vincenzi et al.^17^	KIT exon 9-mutated localized GIST	Retrospective multicenter study	Adjuvant imatinib (variable duration)	Suggested benefit in KIT exon 9-mutated GIST
Napolitano et al.^21^	KIT exon 9-mutated localized GIST	Comparative multicenter study	Adjuvant imatinib versus observation (variable duration)	Evaluated benefit of adjuvant imatinib compared with observation

AIO, Arbeitsgemeinschaft Internistische Onkologie; DFS, disease-free survival; EORTC, European Organisation for Research and Treatment of Cancer; GISTs, gastrointestinal stromal tumors; NIH, National Institutes of Health; OS, overall survival; RFS, recurrence-free survival; SSG, Scandinavian Sarcoma Group.

That said, long-term follow-up data showed that the benefit of post-operative imatinib therapy on OS decreased over time and was limited to patients at the highest risk of relapse. This finding is consistent with the drug’s predominantly cytostatic activity. Even in the 10-year analysis of the SSG-XVIII trial in the 3-year arm, it was demonstrated that half of the patients experienced recurrence, indicating that they had not been cured.^36^ This raises important questions about which patients can be cured with adjuvant therapy and whether it is advisable to extend the duration beyond the previously recommended 3 years. The data from the IMADGIST study indicated that relapse-free survival was longer in patients treated for 6 years of imatinib instead of 3, but there was no OS advantage using the longer course of therapy, at least in the initial analysis.^31^

Another important issue is the duration of adjuvant therapy in the development of resistance. Patients who have undergone R0 resection with no evidence of tumor rupture and who have high-risk GIST are most likely to benefit from 3 years of imatinib therapy if they have a *KIT* exon 11 mutation, a moderate mitotic rate, and a moderate tumor size. Conversely, although patients with tumor rupture, a large tumor size, and a high mitotic rate may stay on treatment for 3 years without progression, many do progress in the subsequent 2 years or more and need to resume imatinib lifelong. The first group of patients has the highest chance of long-term cure. Accordingly, although recurrence is uncommon on imatinib, follow-up imaging can be adjusted to reflect the recurrence risk after stopping imatinib, with closer monitoring during the highest-risk period, 6-18 months after stopping imatinib.^38^ The final data from the SSG-XXIII study, investigating 3 versus 5 years of adjuvant imatinib in the highest-risk GISTs, are also eagerly awaited.

The recently published randomized phase III IMADGIST study investigated whether 6 years of adjuvant imatinib for high-risk localized GISTs improves outcomes compared with 3 years of imatinib.^30^ With a median follow-up of 55 months, the primary endpoint of disease-free survival was significantly better in the 6-year arm (HR 0.40, 95% CI 0.20-0.69, *P* = 0.0008). Nevertheless, no difference in time to imatinib resistance (TTIR) was observed between the two arms, and an OS benefit was not seen in the initial analysis. Conversely, a recent update to the BFR14 study of advanced GIST contradicts the observation of no difference in TTIR in the IMADGIST trial. In BFR14, treatment interruption in patients with ‘advanced’ disease who did not progress was found to be associated with a faster emergence of imatinib resistance and shorter long-term survival.^39^ This finding further suggests that the development of resistance mechanisms depends on whether TKI therapy is administered in the context of minimal residual disease, such as in an adjuvant setting, or in the setting of advanced metastatic disease.

Depending on the risk of recurrence, particular attention should be paid to the individual safety needs of patients and the side effects of imatinib. Interindividual variability in imatinib pharmacokinetics may influence both treatment efficacy and tolerability. Recent data highlighted the potential importance of renal function assessment and therapeutic drug monitoring (TDM) for individualized dosing strategies.^40^ Furthermore, real-world data demonstrated the feasibility of TDM in routine clinical practice and suggested its potential value in selected patients with suboptimal drug exposure or toxicity.^41^ In this regard, TDM could serve as a useful precision measurement method in selected cases, potentially personalizing therapy management. Nevertheless, further prospective studies are needed to confirm its impact on long-term outcomes. Although imatinib presumably is the best-tolerated TKI compared with other approved kinase inhibitors in GISTs, almost 50% of patients discontinued treatment in the 5-year arm of the PERSIST trial.^10^ In this regard, it is crucial for patients to closely monitor any side effects and consider their individual circumstances when interpreting their prognosis.

## Future directions in patient selection for adjuvant treatment

Multiomics methods are increasingly being used to more precisely define patient groups. For example, a retrospective study examined the addition of genomic alterations to risk classification and identified them as potential prognostic biomarkers. Similarly, data on four proteomics-based subtypes could be integrated into the NIH classification to improve individual risk stratification.^42^^,^^43^ Artificial intelligence (AI)-based tools also could help to better assess individual prognosis and the optimal duration of imatinib treatment in the future, as demonstrated in an observational study by Bertsimas et al.^44^

TKI therapies remain insufficient for long-term disease control, and many patients undergoing adjuvant imatinib therapy will develop secondary mutations due to TKI resistance. Thus, understanding resistance mechanisms is essential to enhancing the clinical effectiveness of imatinib. In this context, multiomics and advanced molecular sequencing allow for more precise patient stratification and also detect potential resistance mechanisms and clonal evolution at an early stage.

## Conclusions

Adjuvant treatment of GIST has improved outcomes, but its efficacy is limited to patients at higher risk of relapse. Although 3 years of adjuvant imatinib is the standard treatment for high-risk GIST, recurrence is common. This underscores the importance of better patient selection and personalized treatment duration. Specific *KIT* and *PDGFRA* mutation subtypes have important prognostic and predictive value, and these factors should be incorporated into future risk models. Tumor rupture and intermediate-risk disease are heterogeneous conditions that require individualized decision-making. Advances in molecular profiling, multiomics approaches, and AI-based tools offer a future of more precise risk assessment, personalized therapy, and a better understanding of resistance mechanisms in GISTs.
